# Analyses of Arabidopsis ecotypes reveal metabolic diversity to convert D-amino acids

**DOI:** 10.1186/2193-1801-2-559

**Published:** 2013-10-24

**Authors:** Dirk Gördes, Grit Koch, Kerstin Thurow, Üner Kolukisaoglu

**Affiliations:** Institute of Automation, University of Rostock, Richard-Wagner-Str. 31, D-18119 Rostock, Germany; Center for Life Science Automation (celisca), University of Rostock, Friedrich-Barnewitz-Str. 8, D-18119 Rostock, Germany; Center for Plant Molecular Biology, University of Tübingen, Auf der Morgenstelle 32, 72076 Tübingen, Germany; WESSLING GmbH, Haynauer Straβe 60, 12249 Berlin, Germany

**Keywords:** *Arabidopsis thaliana*, L- and D-amino acids, Chiral separation, High performance liquid chromatography, Mass spectrometry, D-amino acids in soil and plants

## Abstract

For a long time D-enantiomers of proteinogenic L-amino acids were assumed to be physiologically irrelevant for plants. But there is growing evidence that D-amino acids (D-AAs) also fulfil important physiological functions in these organisms. However, the knowledge about the metabolic fate of D-AAs in plants is still scarce and more information about it is needed. To close this gap we established an optimized protocol for the processing and analysis of D- and L-AAs from large numbers of Arabidopsis lines. This included the application of 18 different D-AAs to seedlings, the extraction of free amino acids from the samples and the determination of 16 L-AAs and their corresponding D-enantiomers. To validate our approach we searched for genetic accessions with aberrant amino acid metabolism. Therefore we applied D-AAs on 17 ecotypes of *Arabidopsis thaliana* and analysed their free amino acid contents. These analyses confirmed the suitability of the system for the analysis of large sets of plant samples with enhanced velocity and improved accuracy. Furthermore, the resulting data led to the definition of standard amino acid profiles in response to D-AAs of Arabidopsis seedlings. Within these analyses the ecotype *Landsberg erecta* was found with aberrant metabolic patterns like drastically reduced capabilities to convert different D-AAs to D-alanine and D-glutamate. The presented experimental setup and results of this study offer starting points to dissect the metabolic pathway of D-AAs in plants.

**Electronic supplementary material:**

The online version of this article (doi:10.1186/2193-1801-2-559) contains supplementary material, which is available to authorized users.

## Introduction

D-enantiomers of proteinogenic amino acids were found in different forms in all kingdoms of life. They mostly occur either in peptide bound form or as free D-AAs (for overviews see Fujii 2002; Friedman 2010; Cava et al. 2011). Free D-AAs were detected in bacteria as well as in higher organisms like invertebrates and mammals but also in plants.

There are several reports about the detection of free D-AAs in plants, which are in the focus of the present study (Zenk and Scherf 1963; Brückner and Westhauser 1994,2003; Kullman et al. 1999; Herrero et al. 2007; Gogami et al. 2009). In this organismal group the soil seems to be the major source for these D-AAs as amounts of several milligrams per kilogram of soil can be found (for an overview see Vranova et al. 2012). This assumption was supported by the characterisation of the amino acid transporter AtLHT1 from *Arabidopsis thaliana*. Mutants lacking this protein are apparently impaired in the root uptake of different D-AAs (Svennerstam et al. 2007; Forsum et al. 2008; Gördes et al. 2011). But the extraction of D-AAs from seedlings grown on synthetic media (Brückner and Westhauser 2003; Funakoshi et al. 2008) and the characterisation of D-AA synthesizing enzymes like serine racemase (Fujitani et al. 2006,2007) or D-amino acid aminotransferase (Funakoshi et al. 2008) have also indicated an alternative, endogenous origin for D-AAs.

In contrast to other organisms, the physiological role of D-AAs in plants is still unclear. For a long time the inhibitory effect of several D-AAs on plant growth and their slow degradation were used as arguments that they are detrimental for plants and cannot be utilized as nitrogen sources (Erikson et al. 2004; Forsum et al. 2008; Näsholm et al. 2009). But recently two reports were published which shed new light on the physiological functions of D-AAs for plants. In the first article evidence was presented for the uptake of D-Ala by wheat in defined soil concentrations and its utilisation at rates comparable to other organic or inorganic nitrogen sources (Hill et al. 2011). These findings presented a counterargument that D-AAs could contribute to the nitrogen supply of plants. In the second study it could be shown that in Arabidopsis and tobacco glutamate receptor-like channels (GLRs), plant homologs of animal NMDA receptors, regulate calcium influx and pollen tube growth activated by D-Ser (Michard et al. 2011). This was the first example for a physiological function and the necessity of a D-AA in a plant life cycle.

Nevertheless, the question arises how absorbed or endogenously produced D-AAs are processed by plants. In previous approaches it could be shown that Arabidopsis plants take up and may process many different exogenously applied D-AAs via central metabolic routes (Svennerstam et al. 2007; Gördes et al. 2011). *Arabidopsis thaliana* was chosen to characterise these metabolic routes and determine the essential enzymatic steps and proteins in plants. It was the best suitable species due to the availability of large ecotype and mutant collections and highly resolved genetic information making it the species of choice. Biochemical analysis methods with reasonable input of time and costs were needed for adequately profiling D-AAs. Therefore we established a workflow to analyse the D-AA metabolism in Arabidopsis with increased throughput. We analysed seedlings of 17 ecotypes after application of D-AAs to validate our approach. We were able to verify several previous findings (Gördes et al. 2011) and also to determine three different classes of D-AA metabolisation. An ecotype was found within this screen with aberrant characteristics to process D-AAs: *Landsberg erecta* (Ler-0). The finding of an ecotype with such features was a validation of this approach. Furthermore it provided at least one genetic starting point and a model for the metabolic fate of D-AAs in plants.

## Materials and methods

### Plant material and growth conditions

*Arabidopsis thaliana* plants (ecotypes Bay-0, C24, Col-0, Cvi, Est-1, Kin-0, Ler, Nd-1, Van-0, Shahdara, GOT1, Fr-2, Is-0, Nc-1, Nok-1, HR-5 and Ak-1; for detailed information about these accessions see Lempe et al. 2005) were grown in growth chambers (16 h light, 22°C). Germination of sterile seeds took place in 96-well microtiter plates with one seedling per well in 200–250 μl half-strength MS basal salts (0.5 MS; Murashige and Skoog 1962) with 1% sucrose. The addition of D-AAs to a final concentration of 2 mM took place after 16 days of germination for 20 h. Therefore 18 different D-AAs (D-Ala, D-Arg, D-Asn, D-Asp, D-Gln, D-Glu, D-His, D-Ile, D-Leu, D-Lys, D-Met, D-Phe, D-Pro, D-Ser, D-Thr, D-Trp, D-Tyr, D-Val) were applied. For each application three independent replicates of four seedlings in a pool were used for the extraction of free AAs. Afterwards seedlings were taken out of the medium, thoroughly washed with distilled water and then frozen in liquid nitrogen.

### Amino acid extraction from plant material and determination of D- and L-AAs

Extraction of amino acids from plant material and derivatization of amino acids in the extracts took place as described before (Gördes et al. 2011). Soluble protein concentrations of the plant extracts were determined with the Roti-Quant reagent (Carl Roth GmbH, Karlsruhe, Germany) according to the manufacturer’s protocol. These values were used to normalize the amino acid values of the extracts.

The same reagents were also employed for calibration purposes as given in Gördes et al. (2011). As a difference to this protocol d_8_-L-phenylalanine (Cambridge Isotope Laboratories, USA) was applied as an internal standard instead of phenylglycine. Also the LC/MS analysis has been described in detail elsewhere (Gördes et al. 2011). As differences to this protocol the injection volume was 2.5 μl and the mobile phase compositions were applied according to Table 1. Furthermore the total run time was 12 min (instead of 25), which was divided into 8 time segments to achieve maximum sensitivity. A representative chromatogram is given in Additional file 1: Figure S1. The precursor and product ions for quantifier and qualifier, along with optimized collision energy, are shown in Additional file 1: Table S1.

**Table 1:** **LC gradient**

Chromatographic column			
Time [min]	Flow [mL/min]	Solvent A [%]	Solvent B [%]
0	1	78	22
3	1	75	25
5	1	60	40
8	1	57	43
11.5	1.2	55	45
11.51	1	78	22
Regenerating column			
Time [min]	Flow [mL/min]	Solvent A [%]	Solvent B [%]
0	1	0	100
5	1	0	100
10	1	78	22

Calibration of the assay was calculated using linear or quadratic regression analysis, 1/x weighting of the calibration points, and no forcing through the origin. For each AA, an eleven-point calibration curve (0.5, 1, 2.5, 5, 12.5, 25, 50, 125, 250, 500 and 1,000 μmol/L) was created; the internal standard concentration was 250 μmol/L. A representative calibration graph is shown in Additional file 1: Figure S2 together with transition plots of all measured AAs (Additional file 1: Figures S3, S4 and S5). Equations and correlations of all calibration plots are given in Additional file 1: Table S2. The limit of quantitation (LOQ) for each AA was established by entering a signal-to-noise ratio (SNR) of 10 into the method analysis.

### Statistical analyses of the amino acid measurements

The treatment of the seedlings with 18 D-AAs and the analysis of 32 L- and D-AAs from each sample resulted in the generation of maximally 608 data points for absolute AA content for each analysed ecotype (including controls), each one validated in triplicates. For the comparability of the data the relation of the D-AA treated samples to the untreated ones was calculated and used for further analyses. To assess if D-AA application led to a significant change two criteria were applied: Firstly, just change values below 0.66 and over 1.5 were considered, and secondly a two sided *t*-test was performed with a significance level of p < 0.05. The comprehensive results of all analyses and calculations are summarised in Additional file 2.

## Results

### Improvement of AA detection by analysis methods

The reproducibility and robustness of measurements presented in this study were validated by analyses of plant extracts which were spiked with a mix of 16 different D-AAs (50 μmol/L each). These samples were once split into five equal aliquots, derivatised and measured independently (intraday replicates). In a second series of experiments the same extracts were spiked as mentioned before, derivatised and then measured on five subsequent days (interday replicates). As controls these extracts in unmodified form were derivatised and measured as triplicates.

**Figure 1:**
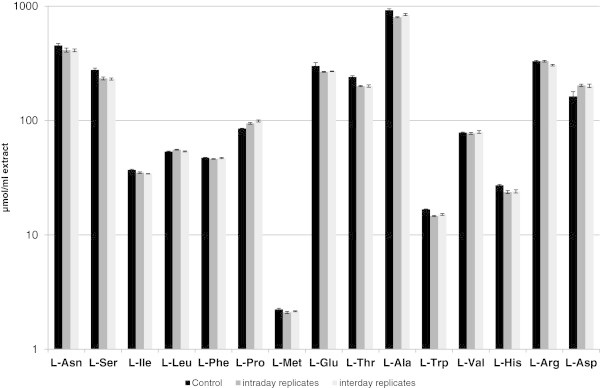
**Measurement of L-AA contents in extracts of** ***A. thaliana*** **(ecotype Ws-2).** For the control measurements the unmodified extract was derivatised and measured thrice. The same extract was then spiked with D-AAs for the intraday and interday measurements. It was then either derivatised and measured five times independently (intraday replicates) or derivatised and afterwards measured on five successive days (intraday replicates). Bars represent the average of measurements (±SD). For further details of the experiment see the main body of the text.

The results of these experiments are summarised in Figure 1 and Table 2. As it can be seen in Figure 1 all 16 targeted proteinogenic L-AAs were detected, whereas L-Gln exceeded the LOQ in all cases. The differences of values between measurements of all other L-AAs were relatively small regardless of the addition of D-AAs. In contrast, only 6 out of 16 D-AAs could be detected in the control extracts. But after spiking the plant extracts with D-AAs they were all detectable in expected amounts. Moreover, the standard deviations of the values in the spiked extracts ranged from 0.5-5.5% (Table 2).

**Table 2:** **Analysis of AA contents in plant extracts without (control measurements) or with addition of 50 μmol D-AAs in intra- and interday measurements (see also main body of the text)**

		D-Asn	D-Ser	D-Ile	D-Leu	D-Phe	D-Pro	D-Met	D-Glu	D-Thr	D-Ala	D-Trp	D-Val	D-His	D-Arg	D-Gln	D-Asp
**Control**	Mean (μmol/L)	1.15	1.76	<LOQ	<LOQ	<LOQ	<LOQ	<LOQ	0.67	2.69	0.69	<LOQ	<LOQ	<LOQ	<LOQ	<LOQ	1.39
**Measurements**	SD in %	8.56	4.10						10.85	1.55	2.20						11.97
**Intraday**	Mean (μmol/L)	51.40	52.39	52.49	53.33	50.49	51.06	53.55	48.56	53.01	53.27	51.56	53.38	47.09	47.35	50.11	53.79
**Measurements**	SD in %	1.93	1.83	1.47	2.21	1.55	1.10	1.41	2.51	1.54	3.85	2.30	1.67	5.56	1.32	1.69	2.10
**Interday**	Mean (μmol/L)	51.85	52.07	51.27	51.49	50.94	53.41	52.75	45.23	53.60	53.42	50.41	52.89	47.43	47.67	50.26	53.47
**Measurements**	SD in %	0.90	1.41	2.33	2.68	2.22	1.99	0.57	2.54	4.51	3.88	1.55	1.78	4.94	2.96	1.49	2.01

To assess the improvement of the novel method compared to the previously described one two ecotypes (Col-0 and C24) were first cultivated and applied with 18 D-AAs as described before. Afterwards the free amino acids were once extracted and then either analysed as described in Gördes et al. (2011) or according to the present report. The results of this comparative approach are summarised in Table 3. As it can be seen from this table, the employment of the new method led to an increased number of analysable data. For the ecotypes Col-0 and C24 the number of non-quantifiable values (meaning either below or beyond the LOQ) dropped from 1057 to 936 and from 978 to 745, respectively. This means that for the first analysed ecotype the percentage of analysable data rose from 42.1% to 48.7% with the improved method. For the second tested ecotype this relation shifted from 48.7% to 56.4%. Table 3 also clarifies that these improvements are not uniform but apply to different analysed AAs in different ecotypes. In only a few cases it could be observed that the old method performed better than the new one, which could not be verified by the analyses of these AAs in C24.

**Table 3:** **Number of quantifiable measurements (out of 54 samples) of particular L- and D-AAs after application of different D-AAs from Col-0 and C24 extracts either analysed with the method from Gördes et al. (** 2011 **, old) or with the improved method presented in this study (new)**

	Col-0				C24			
	Below LOQ		Over LOQ		Below LOQ		Over LOQ	
	***Old***	***New***	***Old***	***New***	***Old***	***New***	***Old***	***New***
**L-Ala**	0	0	50	55	0	0	41	4
**D-Ala**	10	6	0	3	14	6	1	1
**L-Trp**	0	0	0	0	0	0	0	0
**D-Trp**	54	54	0	0	51	51	0	0
**L-Asn**	0	0	48	2	0	0	34	0
**D-Asn**	54	54	1	0	51	51	3	0
**L-Phe**	0	0	0	0	0	0	0	0
**D-Phe**	54	54	3	0	51	51	3	0
**L-Asp**	0	0	0	0	0	0	0	0
**D-Asp**	54	51	0	0	51	51	0	0
**L-Met**	53	0	0	0	50	nd	0	nd
**D-Met**	54	54	0	1	51	nd	0	nd
**L-Gln**	0	0	57	57	0	0	54	53
**D-Gln**	54	54	0	0	51	51	0	0
**L-Val**	0	0	0	0	0	0	0	0
**D-Val**	54	54	0	0	51	51	0	0
**L-Arg**	0	0	31	2	0	0	14	0
**D-Arg**	54	53	1	3	51	51	0	0
**L-Glu**	0	0	0	0	0	0	0	0
**D-Glu**	37	54	0	0	41	18	0	0
**L-His**	0	0	0	0	0	0	0	0
**D-His**	54	54	3	0	51	51	3	0
**L-Ser**	0	0	4	0	0	0	3	0
**D-Ser**	54	54	0	0	51	51	0	0
**L-Ile**	0	0	0	0	0	0	0	0
**D-Ile**	54	54	0	0	51	51	0	0
**L-Thr**	0	0	0	0	0	0	0	0
**D-Thr**	54	54	0	0	51	51	0	0
**L-Pro**	0	0	0	0	0	0	0	0
**D-Pro**	54	54	3	1	51	51	3	0
**L-Leu**	0	0	0	0	0	0	0	0
**D-Leu**	54	54	0	0	51	51	0	0
**SUM**	856	812	201	124	718	687	159	58

### Analysis of free L- and D-AA contents in 17 Arabidopsis ecotypes

After establishing the procedure to determine the AA content in D-AA treated plants, 17 different ecotypes from *Arabidopsis thaliana* were randomly chosen for further analysis. It was one goal of this approach to find ecotypes with aberrant AA patterns after application of D-AAs. Such lines could be used as genetic starting material for the elucidation of the metabolic pathway of D-AAs in plants. This set of ecotypes should underline that this method and approach is able to reach this goal.

The assessment if any given ecotype reacts aberrantly or not in this respect depends on the knowledge and definition of the amino acid profiles in the majority of the ecotypes after application of a particular D-AA. Therefore all those cases were picked out from our analyses in which a specific amino acid significantly increased or decreased in at least four ecotypes after a particular D-AA was applied. After reanalysis of all cases meeting these criteria, three classes of amino acid profile changes in response to D-AA application could be characterised.

In the first reaction class the applied D-AA leads to the change of content of a non-isomeric L-AA. The changes and resulting values, which fall into this class, are summarised in Table 4. As it can be seen in this table, the application of D-AAs led mostly to an increase of the respective L-AA content. The only exception was the conversion D-Glu → L-Ala where a significant decrease was repeatedly observed. Furthermore, Table 4 also reveals that almost all significant changes in this category ranged between 1.5-2.5 fold, whereas only in the ecotype Ak-1 factors of up to 5.9 appeared. Furthermore, the table shows that the majority of effects were caused by the addition of D-Met.

**Table 4:** **Change of L-AA ratio (compared to untreated control plants) after addition of non-corresponding D-AAs (±SD)**

	Ler-0	Nok-1	Van-0	Col-0	Cvi	Nc-1	Kin-0
**D-Glu → L-Ala**	>LOQ	*0.49 ± 0.17*	0.91 ± 0.05	>LOQ	*0.63 ± 0.09*	>LOQ	0.78 ± 0.04
**D-His → L-Met**	1.02 ± 0.14	<LOQ ±	1.21 ± 0.15	1.22 ± 0.14	0.96 ± 0.08	<LOQ	1.06 ± 0.17
**D-Ala → L-Leu**	0.76 ± 0.17	**1.80 ± 0.18**	0.79 ± 0.14	1.06 ± 0.29	1.51 ± 0.30	0.88 ± 0.44	2.16 ± 1.25
**D-Thr → L-Ile**	1.19 ± 0.09	1.20 ± 0.22	*0.61 ± 0.10*	1.13 ±0.08	1.44 ± 0.29	**1.71 ± 0.27**	1.36 ± 0.24
**D-Phe → L-Trp**	1.80 ± 1.96	4.34 ± 1.81	*0.69 ± 0.09*	2.21 ± 0.69	1.26 ± 0.09	0.72 ± 0.06	1.39 ± 0.11
**D-Trp → L-Pro**	**2.41 ± 0.35**	1.64 ± 0.36	0.93 ± 0.17	1.42 ± 0.38	1.10 ± 0.23	1.66 ± 0.31	1.06 ± 0.22
**D-Trp → L-Phe**	1.91 ±0.12	**2.23 ± 0.37**	1.86 ± 0.07	1.77 ± 0.20	1.78 ± 0.26	2.53 ± 0.21	1.99 ± 0.16
**D-Met → L-Leu**	1.27 ± 0.11	1.34 ± 0.42	0.69 ± 0.05	1.50 ± 0.19	1.34 ± 0.25	1.05 ± 0.22	1.27 ± 0.39
**D-Met → L-Ile**	1.20 ± 0.17	1.32 ± 0.70	*0.52 ± 0.03*	1.43 ± 0.26	1.24 ± 0.35	1.11 ± 0.37	1.59 ± 0.24
**D-Met → L-Phe**	1.44 ± 0.35	1.81 ± 0.36	1.17 ± 0.50	1.58 ± 0.63	1.37 ± 0.36	1.14 ± 0.92	**1.77 ± 0.58**
**D-Met → L-Val**	1.39 ± 0.15	1.41 ± 0.37	0.91 ± 0.06	**1.73 ± 0.19**	1.36 ± 0.22	1.11 ± 0.25	1.39 ± 0.20
	**HR-5**	**FR-2**	**Est-1**	**C24**	**Ak-1**	**Got-1**	**Shahdara**
**D-Glu → L-Ala**	>LOQ	>LOQ	*0.67 ± 0.04*	*0.64 ± 0.04*	nd	*0.66 ± 0.08*	nd
**D-His → L-Met**	<LOQ	1.34 ± 0.28	0.94 ± 0.05	nd	1.24 ± 0.33	**1.90 ± 0.27**	**2.74 ± 0.21**
**D-Ala → L-Leu**	0.89 ± 0.05	1.37 ± 0.09	1.40 ± 0.31	**1.76 ± 0.18**	nd	**1.92 ± 0.30**	nd
**D-Thr → L-Ile**	1.34 ± 0.07	1.22 ± 0.11	1.10 ± 0.23	**1.61 ± 0.07**	**4.88 ± 0.54**	1.32 ± 0.21	1.23 ± 0.34
**D-Phe → L-Trp**	1.59 ± 0.36	1.24 ± 0.10	1.67 ± 0.25	**2.04 ± 0.16**	4.65 ± 2.75	**1.95 ± 0.06**	1.02 ± 0.14
**D-Trp → L-Pro**	1.30 ± 0.05	0.85 ± 0.05	0.93 ± 0.13	**1.65 ± 0.10**	**5.08 ± 0.95**	1.67 ± 0.31	1.00 ± 0.10
**D-Trp → L-Phe**	**1.87 ± 0.09**	0.99 ± 0.09	**1.70 ± 0.16**	**1.78 ± 0.27**	**5.91 ± 0.41**	**2.02 ± 0.08**	**1.50 ± 0.15**
**D-Met → L-Leu**	1.34 ± 0.10	**1.74 ± 0.12**	1.46 ± 0.20	**1.95 ± 0.17**	**4.18 ± 0.48**	**1.80 ± 0.06**	**1.67 ± 0.21**
**D-Met → L-Ile**	1.44 ± 0.08	**1.84 ± 0.11**	**1.59 ± 0.29**	**1.67 ± 0.16**	**4.32 ± 0.60**	**1.50 ± 0.12**	**1.78 ± 0.22**
**D-Met → L-Phe**	**1.92 ± 0.09**	1.29 ± 0.11	**1.81 ± 0.03**	**1.59 ± 0.07**	**4.10 ± 0.96**	**1.61 ± 0.20**	**2.52 ± 0.08**
**D-Met → L-Val**	**1.60 ± 0.11**	**1.72 ± 0.20**	**1.54 ± 0.12**	**1.91 ± 0.24**	**3.91 ± 0.27**	**1.76 ± 0.15**	**2.06 ± 0.17**
	**Nd-0**	**Bay-0**	**Is-0**				
**D-Glu → L-Ala**	>LOQ	>LOQ	0.72 ± 0.03				
**D-His → L-Met**	**2.28 ± 0.34**	**1.99 ± 0.13**	1.57 ± 0.47				
**D-Ala → L-Leu**	1.40 ± 0.27	1.31 ± 0.12	**1.72 ± 0.11**				
**D-Thr → L-Ile**	3.14 ± 0.81	**1.94 ± 0.23**	2.59 ± 0.61				
**D-Phe → L-Trp**	**3.43 ± 0.08**	**1.85 ± 0.12**	**2.27 ± 0.04**				
**D-Trp → L-Pro**	**2.13 ± 0.34**	1.47 ± 0.08	2.83 ± 2.09				
**D-Trp → L-Phe**	2.03 ± 0.14	**1.78 ± 0.07**	**1.96 ± 0.05**				
**D-Met → L-Leu**	**1.66 ± 0.10**	1.44 ± 0.11	**1.72 ± 0.06**				
**D-Met → L-Ile**	**1.70 ± 0.05**	**1.58 ± 0.11**	**1.63 ± 0.14**				
**D-Met → L-Phe**	**1.78 ± 0.37**	**1.70 ± 0.10**	**2.06 ± 0.11**				
**D-Met → L-Val**	**2.00 ± 0.19**	**1.93 ± 0.21**	**1.82 ± 0.08**				

Highlighted values: significantly decreased (in italics) or increased (bold) ratio, *nd* not determined.

The second class of changes concerned the increase of an L-AA after application of its enantiomeric D-AA. All such cases of putative racemisation in more than three ecotypes are summarised in Table 5. As it can be seen there in almost all ecotypes, the addition of D-Phe, D-Trp and D-Met led to the increase of the corresponding L-AAs. The putative racemisation of D-His and D-Leu was observed in the majority of ecotypes and this type of change with D-Ile, D-Ser and D-Thr just took place in a smaller subset of analysed ecotypes. Furthermore, the changes in this category were far higher than the ones reported in Table 4. The contents of L-Phe, L-Trp and L-Met rose up to 16, 48 and 230 fold, respectively, after addition of the corresponding D-AAs.

**Table 5:** **Change of L-AA ratio (compared to untreated control plants) after addition of corresponding D-enantiomer (±SD)**

	Ler-0	Nok-1	Van-0	Col-0	Cvi	Nc-1	Kin-0
**D-Ser → L-Ser**	1.10 ± 0.09	1.36 ± 0.46	0.79 + 0.10	1.12 + 0.24	1.01 + 0.06	0.96 + 0.28	1.19 + 0.20
**D-Thr → L-Thr**	0.97 ± 0.07	0.82 ± 0.11	0.82 + 0.08	1.12 + 0.16	1.00 + 0.08	1.21 + 0.20	0.95 + 0.17
**D-Ile → L-Ile**	1.16 ± 0.38	1.33 ± 0.19	0.91 + 0.22	1.65 + 0.40	1.65 + 0.56	1.10 + 0.64	**1.91 + 0.24**
**D-Leu → L-Leu**	1.18 ± 0.21	**2.25 ± 0.37**	0.84 + 0.14	1.38 + 0.27	**2.54 + 0.17**	**1.96 + 0.33**	**1.93 + 0.04**
**D-His → L-His**	0.86 ± 0.13	1.96 ± 0.56	**2.19 + 0.37**	**1.55 + 0.11**	**4.96 + 0.79**	1.97 + 1.23	**2.74 + 0.21**
**D-Phe → L-Phe**	**1.53 ± 0.10**	18.20 ± 6.97	**4.24 + 0.19**	**6.94 + 1.06**	**11.11 + 1.45**	**5.63 + 1.02**	**8.86 + 0.95**
**D-Trp → L-Trp**	**5.02 ± 1.01**	**14.02 ± 3.12**	**5.88 + 1.44**	**12.65 + 3.13**	**10.83 + 2.58**	**13.52 + 1.12**	**8.34 + 1.23**
**D-Met → L-Met**	**1.88 ± 0.14**	27.81 + 10.68	**58.08 + 5.33**	**54.98 + 6.72**	**80.44 + 9.73**	**39.01 + 11.29**	**31.62 + 2.07**
	**HR-5**	**FR-2**	**Est-1**	**C24**	**Ak-1**	**Got-1**	**Shahdara**
**D-Ser → L-Ser**	0.87 ± 0.08	1.05 + 0.18	0.99 + 0.08	1.38 + 0.24	**2.83 + 0.33**	1.41 + 0.12	**1.68 + 0.22**
**D-Thr → L-Thr**	0.97 ± 0.18	0.92 + 0.19	0.92 + 0.19	1.23 + 0.14	**3.40 + 0.33**	1.59 + 0.36	**6.58 + 0.38**
**D-Ile → L-Ile**	1.47 ± 0.17	1.76 + 0.49	1.11 + 0.17	2.04 + 0.39	2.01 + 0.82	**1.83 + 0.19**	**2.02 + 0.15**
**D-Leu → L-Leu**	1.57 ± 0.25	**2.01 + 0.20**	**2.04 + 0.18**	1.72 + 0.29	10.51 + 3.33	nd	**2.08 + 0.14**
**D-His → L-His**	**1.82 ± 0.20**	**3.12 + 0.51**	**2.02 + 0.19**	2.64 + 0.61	6.43 + 3.05	2.87 + 0.50	**3.51 + 0.21**
**D-Phe → L-Phe**	**10.09 ± 2.11**	**3.97 + 0.49**	**8.32 + 1.08**	**6.54 + 0.80**	22.69 + 10.80	6.54 + 0.43	**6.36 + 0.66**
**D-Trp → L-Trp**	**9.54 ± 0.90**	**6.99 + 0.05**	**12.09 + 1.20**	**19.31 + 0.99**	**48.41 + 8.45**	13.66 + 4.89	**1.64 + 0.11**
**D-Met → L-Met**	**34.72 ± 3.19**	**82.44 + 23.83**	**58.78 + 10.02**	nd	**230.89 + 41.96**	76.07 + 27.32	**62.85 + 9.77**
	**Nd-0**	**Bay-0**	**Is-0**				
**D-Ser → L-Ser**	**1.52 ± 0.12**	**1.57 + 0.13**	**1.66 + 0.15**				
**D-Thr → L-Thr**	1.43 ± 0.24	**1.56 + 0.06**	**1.57 + 0.11**				
**D-Ile → L-Ile**	**2.78 ± 0.30**	**1.89 + 0.29**	**1.78 + 0.06**				
**D-Leu → L-Leu**	**2.27 ± 0.43**	1.66 + 0.26	**1.83 + 0.08**				
**D-His → L-His**	**6.66 ± 0.56**	**3.78 + 0.62**	**4.28 + 0.92**				
**D-Phe → L-Phe**	**16.64 ± 0.71**	**5.67 + 0.38**	**12.80 + 1.09**				
**D-Trp → L-Trp**	**44.75 ± 7.76**	**9.39 + 0.32**	21.48 + 9.13				
**D-Met → L-Met**	**185.20 ± 9.66**	**73.48 + 12.75**	**88.09 + 13.61**				

Bold values: significantly increased ratio, *nd* not determined.

In the third class of changes, which were also observed in Gördes et al. (2011), D-Ala and D-Glu accumulated after addition of most D-AAs in almost all characterised ecotypes (Additional file 1: Tables S3 and S4). The accumulation of D-Ala and D-Glu occurred in almost all tested ecotypes, whereas the accumulation of D-Ala was much stronger and more consistent than the one of D-Glu. Almost all applied D-AAs led to increased D-Ala concentrations in the plants, whereupon D-Met had again the strongest impact with changes of up to 788 fold (Additional file 1: Table S3). The influence on the accumulation of D-Glu was less pronounced, and in this case application of D-Ala was responsible for the strongest effects (Additional file 1: Table S4). It is noteworthy, that most of these relative changes have to be considered as minimum changes, because in several ecotypes the measured concentrations of D-Ala and D-Glu were below the LOQ in the control plants. In these cases the values were set to the lower limit of our system (0.5 nmol/ml). This means that effectively the relative changes might have been even higher.

### The ecotype *Landsberg erecta* shows aberrant reactions against D-AAs

**Figure 2:**
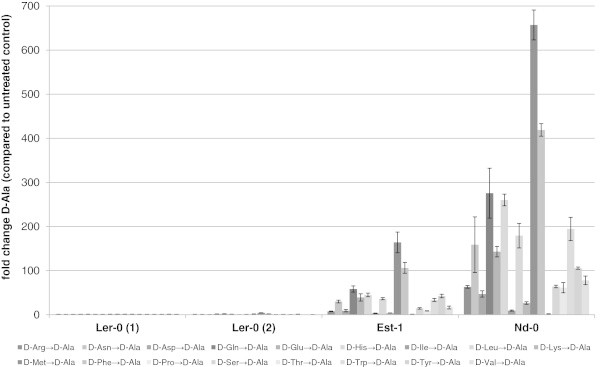
**Accumulation of D-Ala in particular Arabidopsis accessions after 20 h of growth in medium supplemented with different D-AAs.** As examples for the plasticity of conversion of D-AAs to D-Ala in *A. thaliana* results for Est-1, Nd-0 and two independent seed supplies of the ecotype Ler-0 are shown. In the diagram the ratio of accumulated D-Ala in plant extracts supplemented with D-AAs compared to untreated controls is shown. Bars represent the average of measurements (±SD) from three samples with four plants in each sample.

**Figure 3:**
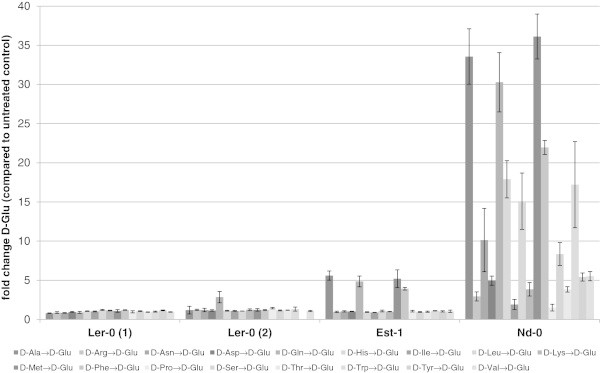
**Accumulation of D-Glu in particular Arabidopsis accessions after 20 h of growth in medium supplemented with different D-AAs.** As examples for the plasticity of conversion of D-AAs to D-Glu in *A. thaliana* results for Est-1, Nd-0 and two independent seed supplies of the ecotype Ler-0 are shown. In the diagram the ratio of accumulated D-Glu in plant extracts supplemented with D-AAs compared to untreated controls is shown. Bars represent the average of measurements (±SD) from three samples with four plants in each sample.

The main purpose of the present study was to test if ecotypes with aberrant metabolism of D-AAs can be found by this method. In the presented study such an ecotype could be identified. It was previously shown (Additional file 1: Tables S3 and S4) that almost all ecotypes reacted on D-AAs. But the ecotype *Landsberg erecta* (Ler-0) formed an exception. This ecotype showed no accumulation of either D-Ala or of D-Glu after exogenous application of any D-AA. To verify this aberrant response on D-AAs as well as to characterise it in more detail, a new batch of seeds of Ler-0 was germinated, treated with D-AAs and analysed as in the first set of experiments. The results of these experiments are summarised in the Figures 2 and 3.

In Figure 2 the accumulation of D-Ala is illustrated in both sets of analyses with Ler-0 compared with Est-1 and Nd-0. The latter ecotypes exhibited the smallest, and respectively, the largest changes of D-Ala in response to exogenous D-AAs (apart from Ler-0). In this figure it becomes obvious that in all tests the reactions against any D-AA in Ler-0 were neglectable, whereas even in Est-1, the majority of D-AAs caused an increase of D-Ala between 7–163 fold. The trend is less pronounced when it comes to the accumulation of D-Glu. In Figure 3 it can be seen that this accumulation can differ between the selected ecotypes. As one extreme, Nd-0 showed significant responses against most of the applied D-AAs. On the other side, Est-1 revealed accumulation of D-Glu in response just to four D-AAs, and to a lesser extent than Nd-0. Nevertheless, the application of D-AAs caused no reproducible increase of D-Glu content in Ler-0.

**Figure 4:**
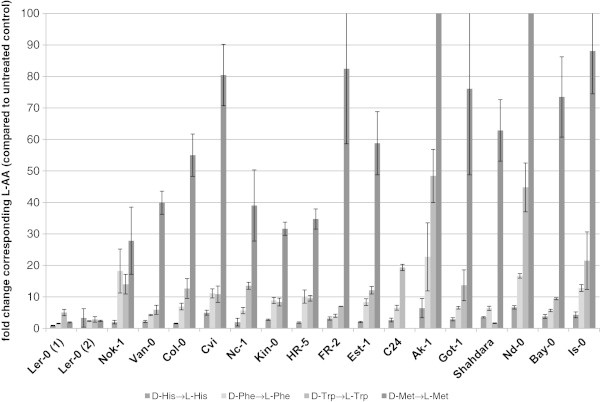
**Conversion of D-His, D-Phe, D-Trp and D-Met to their corresponding L-AAs in different Arabidopsis ecotypes.** Seedlings of all tested ecotypes (including two independent seed supplies of the ecotype Ler-0) were treated for 20 h with D-His, D-Phe, D-Trp and D-Met. In the diagram the ratio of the corresponding L-AAs in plant extracts supplemented with these D-AAs compared to untreated controls is shown. Bars represent the average of measurements (±SD) from three samples with four plants in each sample.

Hence, the aberrant responses of Ler-0 were not just limited to a missing accumulation of D-Ala and D-Glu but also the formation of L-AAs after addition of the corresponding D-AAs (Table 5) was affected in this ecotype. This effect is illustrated in Figure 4 for the putative racemisations of His, Trp, Phe and Met for all analysed ecotypes. It becomes obvious that at least the concentrations of L-Trp and L-Met increased in almost all analysed ecotypes after addition of the corresponding D-AA. In contrast, Ler-0 did neither show reproducibly these responses nor a putative racemisation of any other D-AA (Figure 4 and Table 5).

## Discussion

In this study a versatile method was presented to analyse the contents of D- and L-AAs in a large number of plant samples. In a proof of principle experiment with 17 ecotypes of *Arabidopsis thaliana* general metabolic reactions of plants against D-AAs could be either verified or determined for the first time with this method. The major goal of this approach was the establishment of a technique to identify genetic lines with aberrant metabolism of D-AAs. The identification of Ler-0 as an ecotype with a lack to convert various D-AAs either to their corresponding L-AAs or to D-Ala and D-Glu can be accounted as a successful application of this method. Furthermore, the identification of an ecotype with aberrant D-AA metabolism provides first insights into its mechanisms.

A variety of methods and protocols have been published to detect and analyse D- and L-AAs from plant extracts (Ali et al. 2006; Brückner and Westhauser 1994,2003; Herrero et al. 2007). In a previous study (Gördes et al. 2011) a method was described, which represented an alternative to existing methods specifically developed for the analysis of Arabidopsis plant material. The described procedure in this study represents an improvement to this method. As it was shown above the portion of quantifiable data increased with the present method (Table 3). The improved range of quantitation might explain this effect. The upper LOQ for the present study was determined at 1,000 μmol/L, with a lower LOQ of 0.5 μmol/L (see Additional file 1: Table S2). In Gördes et al. (2011) the range of detection was between 1.25/5-500 μmol/L. This means, that an improvement of sensitivity from 5–20 fold was achieved by the optimised system described in the present study. Furthermore it has been shown in preliminary studies that this method is applicable to other plant species ranging from mosses over monocots to different dicots (data not shown). Although final validation of the latter is still pending this would point to a general applicability of this procedure for many different plant species.

Besides the extended concentration range additional important improvements of the described method are the reduction of the overall analysis time per sample and the exceptionally long-term stability. Compared to the chromatographic method in Gördes et al. (2011), the time was cut by half (12 min instead of 25 min). Due to this time reduction up to 120 measurements were possible within 24 hours. For this effect, an optimised mobile phase gradient, downsized column dimensions, and the implementation of an alternating column regeneration (ACR, Agilent Technology) mode were necessary. With ACR, a system of two identical columns allows the accomplishment of the complete cleaning and equilibration process in parallel to the chromatographic measurement. Furthermore, in combination with a reduced injection volume the column lifetime could be increased by factor of 10, up to several thousand measurements.

The major goal of the presented work was the analysis of different accessions of *A. thaliana* to identify ecotypes with aberrant responses against D-AAs. Therefore a large number of AA measurements were performed. A general observation over all measurements was that the vast majority of changes in all accessions in response to exogenous D-AAs represented increases of AA levels as only few decreases were found (Tables 4, 5, Additional file 1: Tables S3 and S4). This supports the hypothesis that plants acquire and metabolise D-AAs as nitrogen sources (Hill et al. 2011, Vranova 2012).

The large number of ecotypes, treatments and measurements may also have contributed to answer a question, which arose in Gördes et al. (2011). There it was asked why just D-His, D-Met, D-Phe and D-Trp led to putative racemisations. In the light of the actual results it can be said that D-Ile, D-Leu, D-Ser and D-Thr also cause a similar effect. The conversion rates are more moderate and do not appear as regularly as for the first four D-AAs (Table 5). But the putatively racemised D-AAs do not seem to be limited to D-His, D-Met, D-Phe and D-Trp. Currently it cannot be said if other D-AAs also could cause putative racemisations in other ecotypes. Furthermore, the problem remains that for most of these 8 D-AAs no racemase from plants is known. A plant alanine racemase had been characterised, but is still not identified (Ono et al. 2006 Nishimura et al. 2007). A serine racemase however has been isolated and characterised from different plant species. This enzyme possesses an additional L-serine dehydratase activity, which would possibly explain the moderate conversion rates (Fujitani et al. 2006, 2007, Gogami et al. 2009).

One prerequisite to identify plant accessions with aberrant reactions against D-AAs was the observation and definition of common reactions in the analysed plants. As an outcome of this study three major classes of reactions in response to applied D-AAs were defined (see Results). It was interesting in this respect that each class of reactions was not just specified by the type of reaction but also by extent and distribution. In the first reaction class (D-AA → L-non-enantiomer) the rate of reaction at 1.5-5 fold was relatively small and in some ecotypes just one particular reaction of this class was observed, whereas in other ecotypes almost all of them appeared. The relatively low rate of changes and their facultative appearance in different ecotypes might be a reason why this reaction class was not reported in previous studies (Chen et al. 2010; Gördes et al. 2011). In comparison the reaction rates of the second class (D-AA → L-enantiomer) and the third class of reactions (D-AA → D-Ala/Glu) were much higher with increases up to >700 fold (D-Met → D-Ala; Additional file 1: Table S3). Only the conversion of D-AA → D-Ala could be observed in almost all ecotypes and after all treatments. Instead putative racemisations and D-AA → D-Glu conversions were found often, but not in such regular manner. Especially the high rates in the latter two reaction classes may be an indicator that these reactions are directly catalysed, whereas the observed conversions summarised in the first class of reaction are indirect changes in response to D-AA application and therefore of a rather secondary type.

The identification of the ecotype *Landsberg erecta* as an accession with drastically reduced abilities to metabolise exogenously applied D-AAs can be accounted as a proof of the presented concept for the investigation of plant D-AA metabolism. As one example there is still the unanswered question about the catalytic mechanisms leading to the formation of enantiomers and specific D-AAs. Vranova et al. (2012) suggested racemisation, deamination or transamination as putative reactions being responsible for the observed phenomena. The aberrant responses of *Landsberg erecta* may serve as a genetic starting point to answer the posed question.

**Figure 5:**
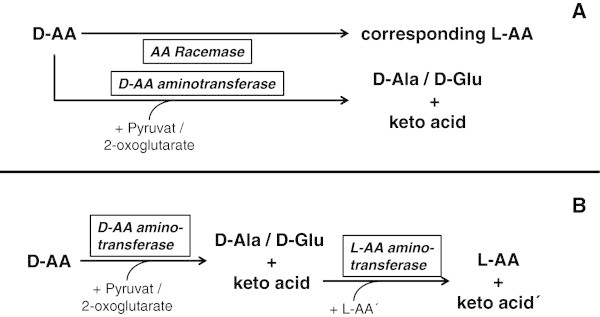
**Possible ways of D-AA conversion in** ***Arabidopsis thaliana*** **.** In the first scenario **(A)** the applied D-AAs are metabolised by a putative racemase and a D-AA aminotransferase. In the second scenario **(B)** exogenously applied D-AAs are metabolised under the assumption that in the first step these D-AAs are transaminated to D-Ala/D-Glu by a putative D-AA aminotransferase. The remaining keto acid is then transaminated by a corresponding L-AA aminotransferase.

All applied D-AAs were detectable in reasonable amounts in the analysed plant extracts (Additional file 2). Due to this fact, defective import of D-AAs, as observed in the AA transporter mutant *lht1* (Gördes et al. 2011), can be excluded. Instead a defect in D-AA metabolism has to be assumed. In principle there are two possible genetic scenarios which would explain the results in Ler-0. Both scenarios are schematised in Figure 5. The assumption in the first model is that at least two enzymes are responsible for the effects in Ler-0 (Figure 5A). An unspecific AA racemase leads to the conversion of D-AAs to their corresponding enantiomers, and a D-AA aminotransferase is responsible for the evolution of D-Ala and D-Glu. This means that at least two genes are mutated in Ler-0. Furthermore, this model requires an unspecific AA racemase. But such a single enzyme has not been shown yet (for an overview about AA racemases see Conti et al. 2011). This would mean that probably more than one candidate racemase gene got lost in Ler-0 to explain the presented results. This makes this model less probable than the second one (Figure 5B), which proposes just the loss of function of a single enzyme, a D-AA aminotransferase of primary responsibility. According to this scenario the observed putative racemisation in almost all ecotypes would then be the result of secondary L-AA aminotransferase reactions using the remaining keto acid from the D-AA aminotransferase reaction with an available L-AA. There are several highly expressed L-AA aminotransferases in Arabidopsis cells which are responsible for a number of reactions in primary and secondary metabolism. A loss of such a primary D-AA aminotransferase function in Ler-0 would explain the results. The characterisation of a D-AA aminotransferase has been reported previously (Funakoshi et al. 2008) and it has been shown that such enzymes are expressed in Arabidopsis, but it is not known yet if this enzyme or putative functional homologs can act in the proposed way. Nevertheless, the aberrant reaction of Ler-0 points to a defect in D-AA aminotransferase reaction and makes this reaction also most probable as the primary cause for D-AA conversion in the list of candidates of Vranova et al. (2012).

Furthermore, the presented data raise the question about the physiological significance of D-AAs in plants. From previous studies it is known that not all D-AAs have detrimental effects on Arabidopsis plants and that some of them (e.g. D-Val, D-Lys, D-Ile) even promote plant growth (Erikson et al. 2004 Chen et al. 2010; Gördes et al. 2011). Recently it was shown that wheat plants are able to take up and utilise D-Ala (Hill et al. 2011). This was the first time it was shown that plants are capable to make use of D-AAs as a source of nitrogen supply. The results presented in this study revealed, beside the aberrant reaction of Ler-0, gradually differing amino acid conversions in the analysed ecotypes. These observations might reflect the varying capabilities of different accessions to utilise D-AAs. Another possibility for the different amino acid profiles in the ecotypes might be their different capacities to degrade D- and L-AAs leading to differential accumulation of amino acids.

Being a source of nitrogen may be one function of D-AAs in plants. As previously shown for other organisms like bacteria or mammals particular D-AAs like D-Ala, D-Glu or D-Ser also act as regulatory molecules (for overviews see Friedman 2010; Cava et al. 2011). In Arabidopsis such a relationship was recently shown for the first time by the influence of D-Ser on pollen tube development. Furthermore, it was revealed that D-serine racemase is involved in D-Ser mediated signal transduction (Michard et al. 2011). In this respect it would be interesting to compare pollination of the different accessions with their capability to metabolise D-Ser. Nevertheless, the role of D-Ser and possibly other D-AAs as regulatory molecules and the involvement of a D-AA metabolising enzyme in a D-AA influenced developmental process point out the importance of the homeostasis of D-AAs in plant life cycle.

The method presented in this report for amino acid profiling might contribute to addressing some of the questions given above due to its potential for the analysis of large numbers of plant samples. The processing and analysis of 17 Arabidopsis ecotypes confirmed the capabilities of the method. The wide variety of amino acid profiles in these accessions, especially the identification of Ler-0 as an ecotype with aberrant reactions against D-AAs, revealed a great plasticity of *A. thaliana* to metabolise these molecules. Further genetic and molecular analyses of D-AA metabolism of the given ecotypes, additionally of knock out mutants of candidate genes, but also of other plant species, should result in a larger insight into this metabolic pathway. Detailed comparison of amino acid profiles and morphological and physiological phenotyping of these plants in response to D-AAs should also answer the question if D-AAs have additional regulatory functions or act primarily as nitrogen sources.

## Electronic supplementary material

Additional file 1: Table S1: Amino Acids Multiple Reaction Monitoring (MRM) Acquisition Parameters. **Table S2:** Slope, Correlation, and Limit of Quantitation for D- and L-AAs. **Table S3:** Change of D-Ala ratio (compared to untreated control plants) after addition of D-AAs. **Table S4:** Change of D-Glu ratio (compared to untreated control plants) after addition of D-AAs. **Figure S1:** Total ion chromatogram (TIC) showing up 34 analytes on the basis of 68 MRMs within 8 time segments (TS). **Figure S2:** Representative calibration plot for D-Arg. **Figure S3:** Transitions for AAs D-His, L-His, L-Asn, D-Asn, L-Ser, D-Ser, L-Ala, D-Ala, L-Met, D-Met, L-Ile, L-Leu, D-Ile, and D-Leu. **Figure S4:** Transitions for AAs L-Asp, D-Asp, L-Thr, D-Thr, L-Glu, D-Glu, Gly, L-Val, D-Val, L-Trp, and D-Trp. **Figure S5:** Transitions for AAs D-Arg, L-Arg, L-Gln, D-Gln, L-Pro, D-Pro, L-Phe d_8_ (internal standard), L-Phe, and D-Phe. (DOCX 323 KB)

Additional file 2: **Contents and ratio changes (compared to untreated control plants) of L- and D-AAs after treatment with different D-AAs in various Arabidopsis accessions.** (XLSX 586 KB)
