# Supplementary material for: Analyses of Arabidopsis ecotypes reveal metabolic diversity to convert D-amino acids
Source: Springerplus. 2013 Oct 24;2:559. doi: 10.1186/2193-1801-2-559 (PMC3825070; doi:10.1186/2193-1801-2-559)

**Additional file 1 for Gördes et al.**

**Table S1:** Amino Acids Multiple Reaction Monitoring (MRM) Acquisition Parameters

| **Analyte** | **TS** | **RT [min]** | **Ion** | **Precursor ion** | **Product ion** | **Fragmentor [V]** | **CE [V]** |
| --- | --- | --- | --- | --- | --- | --- | --- |
| D-His | 1 | 2.00 | Quantifier | 436.1 | 302.2 | 140 | 10 |
|  |  |  | Qualifier | 436.1 | 284.1 | 140 | 12 |
| L-His | 1 | 2.28 | Quantifier | 436.1 | 302.2 | 140 | 10 |
|  |  |  | Qualifier | 436.1 | 284.1 | 140 | 12 |
| D-Arg | 1 | 2.52 | Quantifier | 455.1 | 130.1 | 135 | 23 |
|  |  |  | Qualifier | 455.1 | 202.1 | 135 | 28 |
| L-Arg | 1 | 2.66 | Quantifier | 455.1 | 130.1 | 135 | 23 |
|  |  |  | Qualifier | 455.1 | 202.1 | 135 | 28 |
| L-Asn | 2 | 3.96 | Quantifier | 413.1 | 368.1 | 110 | 3 |
|  |  |  | Qualifier | 413.1 | 322.1 | 110 | 7 |
| D-Asn | 2 | 4.16 | Quantifier | 413.1 | 368.1 | 110 | 3 |
|  |  |  | Qualifier | 413.1 | 322.1 | 110 | 7 |
| L-Ser | 3 | 4.52 | Quantifier | 386.1 | 341.1 | 105 | 2 |
|  |  |  | Qualifier | 386.1 | 296.1 | 105 | 2 |
| L-Gln | 3 | 4.55 | Quantifier | 427.1 | 382.0 | 100 | 2 |
|  |  |  | Qualifier | 427.1 | 336.2 | 100 | 6 |
| L-Asp | 3 | 4.70 | Quantifier | 414.1 | 369.2 | 120 | 3 |
|  |  |  | Qualifier | 414.1 | 324.1 | 120 | 3 |
| L-Thr | 3 | 4.77 | Quantifier | 400.1 | 355.1 | 100 | 4 |
|  |  |  | Qualifier | 400.1 | 309.1 | 100 | 6 |
| D-Gln | 3 | 4.77 | Quantifier | 427.1 | 382.0 | 100 | 2 |
|  |  |  | Qualifier | 427.1 | 336.2 | 100 | 6 |
| D-Ser | 3 | 5.02 | Quantifier | 386.1 | 341.1 | 105 | 2 |
|  |  |  | Qualifier | 386.1 | 296.1 | 105 | 2 |
| L-Glu | 3 | 5.13 | Quantifier | 428.1 | 383.2 | 100 | 3 |
|  |  |  | Qualifier | 428.1 | 338.2 | 100 | 6 |
| D-Asp | 3 | 5.17 | Quantifier | 414.1 | 369.2 | 120 | 3 |
|  |  |  | Qualifier | 414.1 | 324.1 | 120 | 3 |
| D-Glu | 4 | 5.62 | Quantifier | 428.1 | 383.2 | 100 | 3 |
|  |  |  | Qualifier | 428.1 | 338.2 | 100 | 6 |
| Gly | 4 | 5.65 | Quantifier | 356.1 | 311.0 | 100 | 3 |
|  |  |  | Qualifier | 356.1 | 266.0 | 100 | 3 |
| L-Ala | 4 | 5.83 | Quantifier | 370.1 | 325.2 | 120 | 3 |
|  |  |  | Qualifier | 325.1 | 205.1 | 125 | 9 |
| L-Pro | 4 | 6.06 | Quantifier | 396.1 | 351.2 | 140 | 2 |
|  |  |  | Qualifier | 396.1 | 231.1 | 140 | 19 |
| **Analyte** | **TS** | **RT [min]** | **Ion** | **Precursor ion** | **Product ion** | **Fragmentor [V]** | **CE [V]** |
| D-Thr | 4 | 6.10 | Quantifier | 400.1 | 355.1 | 100 | 4 |
|  |  |  | Qualifier | 400.1 | 309.1 | 100 | 6 |
| D-Pro | 5 | 6.65 | Quantifier | 396.1 | 351.2 | 140 | 2 |
|  |  |  | Qualifier | 396.1 | 231.1 | 140 | 19 |
| L-Met | 5 | 6.75 | Quantifier | 430.1 | 385.2 | 100 | 3 |
|  |  |  | Qualifier | 430.1 | 340.1 | 100 | 3 |
| D-Ala | 5 | 6.85 | Quantifier | 370.1 | 325.2 | 120 | 3 |
|  |  |  | Qualifier | 325.1 | 205.1 | 125 | 9 |
| L-Val | 5 | 6.95 | Quantifier | 398.1 | 352.2 | 100 | 2 |
|  |  |  | Qualifier | 398.1 | 233.1 | 100 | 14 |
| L-Trp | 6 | 7.56 | Quantifier | 485.1 | 188.1 | 120 | 4 |
|  |  |  | Qualifier | 485.1 | 146.1 | 120 | 19 |
| L-Ile | 6 | 7.89 | Quantifier | 412.1 | 366.2 | 110 | 3 |
|  |  |  | Qualifier | 412.1 | 309.1 | 110 | 5 |
| L-Phe d_8_ | 6 | 7.89 | Quantifier | 454.1 | 408.1 | 100 | 2 |
|  |  |  | Qualifier | 454.1 | 236.0 | 100 | 19 |
| L-Phe | 6 | 7.94 | Quantifier | 446.1 | 400.2 | 100 | 2 |
|  |  |  | Qualifier | 446.1 | 235 | 100 | 19 |
| L-Leu | 6 | 8.05 | Quantifier | 412.1 | 366.2 | 110 | 3 |
|  |  |  | Qualifier | 412.1 | 309.1 | 110 | 5 |
| D-Met | 7 | 8.56 | Quantifier | 430.1 | 385.2 | 110 | 3 |
|  |  |  | Qualifier | 430.1 | 340.1 | 110 | 3 |
| D-Trp | 7 | 9.04 | Quantifier | 485.1 | 188.1 | 120 | 4 |
|  |  |  | Qualifier | 485.1 | 146.1 | 120 | 19 |
| D-Val | 7 | 9.38 | Quantifier | 398.1 | 352.2 | 100 | 2 |
|  |  |  | Qualifier | 398.1 | 233.1 | 100 | 14 |
| D-Phe | 8 | 10.39 | Quantifier | 446.1 | 400.2 | 100 | 2 |
|  |  |  | Qualifier | 446.1 | 235 | 100 | 19 |
| D-Ile | 8 | 11.17 | Quantifier | 412.1 | 366.2 | 110 | 3 |
|  |  |  | Qualifier | 412.1 | 309.1 | 110 | 5 |
| D-Leu | 8 | 11.36 | Quantifier | 412.1 | 366.2 | 110 | 3 |
|  |  |  | Qualifier | 412.1 | 309.1 | 110 | 5 |

**Table S2**: Slope, Correlation, and Limit of Quantitation for D- and L-AAs

| **Analyte** | **Equation** | **Correlation (r^2^)** | **LOQ [µmol/L]** |
| --- | --- | --- | --- |
| Gly | y = 0.5333x – 0.00084 | 0.9997 | 0.5 |
| L-Ala | y = -0.0183x^2^ + 0.5427x – 0.00009 | 0.9971 | 0.5 |
| D-Ala | y = -0.0690x^2^ + 0.9811x – 0.00003 | 0.9974 | 0.5 |
| L-Arg | y = 0.4297x – 0.00010 | 0.9991 | 0.5 |
| D-Arg | y = 0.6140x – 0.00023 | 0.9999 | 0.5 |
| L-Asn | y = -0.0512x^2^ + 0.9514x + 0.00005 | 0.9998 | 0.5 |
| D-Asn | y = -0.0363x^2^ + 0.7413x + 0.00004 | 0.9999 | 0.5 |
| L-Asp | y = 0.3400x – 0.00009 | 0.9868 | 0.5 |
| D-Asp | y = 0.3953x – 0.00015 | 0.9997 | 0.5 |
| L-Gln | y = -0.0078x^2^ + 0.1427x – 0.00003 | 0.9996 | 0.5 |
| D-Gln | y = -0.0146x^2^ + 0.2002x + 0.00006 | 0.9997 | 0.5 |
| L-Glu | y = 0.0024x^2^ + 0.4021x – 0.00006 | 0.9992 | 0.5 |
| D-Glu | y = 0.0036x^2^ + 0.5133x – 0.00028 | 0.9810 | 0.5 |
| L-His | y = 0.0141x^2^ + 0.5010x – 0.00003 | 0.9974 | 0.5 |
| D-His | y = 0.0195x^2^ + 0.4008x – 0.00002 | 0.9975 | 0.5 |
| L-IIe | y = 2.3668x – 0.00037 | 0.9994 | 0.5 |
| D-IIe | y = 3.4029x – 0.00242 | 0.9997 | 0.5 |
| L-Leu | y = 2.3282x – 0.00066 | 0.9986 | 0.5 |
| D-Leu | y = 2.1001x – 0.00087 | 0.9998 | 0.5 |
| L-Met | y = 0.5665x – 0.00015 | 0.9925 | 0.5 |
| D-Met | y = 0.3422x – 0.00006 | 0.9893 | 0.5 |
| L-Phe | y = -0.0098x^2^ + 0.9326x – 0.00009 | 0.9984 | 0.5 |
| D-Phe | y = 0.0352x^2^ + 1.0676x – 0.00066 | 0.9975 | 0.5 |
| L-Pro | y = 0.0257x^2^ + 0.2139x – 0.00012 | 0.9985 | 0.5 |
| D-Pro | y = -0.0278x^2^ + 0.6100x + 0.00004 | 0.9986 | 0.5 |
| L-Ser | y = -0.0096x^2^ + 0.5155x – 0.00015 | 0.9996 | 0.5 |
| D-Ser | y = -0.0161x^2^ + 0.6178x – 0.00001 | 0.9998 | 0.5 |
| L-Thr | y = 0.1902x – 0.00001 | 0.9980 | 0.5 |
| D-Thr | y = -0.0178x^2^ + 0.5445x + 0.00006 | 0.9966 | 0.5 |
| L-Trp | y = -0.1302x^2^ + 1.4719x + 0.00014 | 0.9993 | 0.5 |
| D-Trp | y = -0.1036x^2^ + 1.3148x + 0.00026 | 0.9995 | 0.5 |
| L-Val | y = -0.0772x^2^ + 1.1383x + 0.00267 | 0.9997 | 0.5 |
| D-Val | y = -0.0467x^2^ + 1.2854x – 0.00039 | 0.9997 | 0.5 |

**Table S3**: Change of D-Ala ratio (compared to untreated control plants) after addition of D-AAs

|  | **Ler-0** | **Col-0** | **Bay-0** | **Cvi** | **Est-1** | **Kin-0** | **Nd-0** | **Van-0** | **Got-1** | **FR-2** | **Is-0** | **Nc-1** | **Nok-1** | **HR-5** | **C24** | **Shahdara** | **Ak-1** |
| --- | --- | --- | --- | --- | --- | --- | --- | --- | --- | --- | --- | --- | --- | --- | --- | --- | --- |
| **D-Ala→D-Ala** | 608.47 | >LOQ | >LOQ | 1446.89 | 389.24 | 1649.60 | >LOQ | 754.96 | 1143.12 | >LOQ | 1093.69 | 730.71 | 1651.08 | 1032.79 | >LOQ | nd | nd |
| **D-Arg→D-Ala** | 0.87 | 39.15 | 28.40 | 10.39 | 7.58 | 13.76 | 63.15 | 4.96 | 8.63 | 24.30 | 25.26 | 16.17 | 4.51 | 6.16 | 12.01 | nd | nd |
| **D-Asn→D-Ala** | 0.83 | 96.96 | 121.73 | 68.65 | 29.94 | 73.75 | 158.74 | 32.26 | 33.79 | 170.74 | 57.65 | 33.62 | 43.56 | 47.67 | 66.93 | nd | nd |
| **D-Asp→D-Ala** | 0.95 | 21.34 | 33.43 | 35.47 | 9.16 | 30.56 | 47.09 | 6.57 | 7.77 | 55.49 | 23.80 | 4.41 | 26.67 | 9.86 | 37.35 | nd | nd |
| **D-Gln→D-Ala** | 0.89 | 130.16 | 147.68 | 162.66 | 58.24 | 151.73 | 275.60 | 49.68 | 80.74 | 152.71 | 94.35 | 46.18 | 115.54 | 74.06 | 105.43 | 130.78 | 216.01 |
| **D-Glu→D-Ala** | 0.93 | 140.49 | 109.07 | 75.36 | 39.16 | 59.97 | 143.04 | 46.41 | 48.08 | 146.51 | 94.52 | 29.05 | 68.77 | 52.98 | 76.27 | 73.30 | 87.48 |
| **D-His→D-Ala** | 1.05 | 147.25 | 164.61 | 95.67 | 44.90 | 114.64 | 260.10 | 62.43 | 67.97 | 133.62 | 110.14 | 71.05 | 59.02 | 74.62 | 75.67 | 106.70 | 123.16 |
| **D-Ile→D-Ala** | 1.02 | 9.01 | 6.21 | 8.38 | 3.11 | 8.17 | 9.22 | 2.80 | 2.29 | 6.20 | 3.75 | 7.39 | 3.19 | 2.62 | 2.61 | 6.54 | 6.91 |
| **D-Leu→D-Ala** | 1.21 | 82.05 | 73.65 | 70.65 | 36.29 | 73.99 | 179.26 | 40.84 | nd | 71.09 | 49.81 | 70.25 | 103.22 | 55.00 | 72.31 | 67.12 | 350.65 |
| **D-Lys→D-Ala** | 1.13 | 13.32 | 15.03 | 6.61 | 3.86 | 7.62 | 26.52 | 4.93 | 3.94 | 7.96 | 4.53 | 29.71 | 4.01 | 5.36 | 3.34 | 7.73 | 49.58 |
| **D-Met→D-Ala** | 1.08 | 634.67 | 294.85 | 241.17 | 163.97 | 234.71 | 656.91 | 192.50 | 230.11 | 388.80 | 251.04 | 247.17 | 268.38 | 276.56 | 288.72 | 233.22 | 788.79 |
| **D-Phe→D-Ala** | 1.20 | 350.26 | 194.83 | 152.38 | 106.10 | 184.36 | 418.85 | 88.42 | 127.83 | 184.51 | 183.84 | 136.71 | 350.70 | 167.24 | 207.85 | 116.71 | 382.00 |
| **D-Pro→D-Ala** | 0.97 | 0.79 | 0.03 | 0.80 | 1.05 | 1.58 | 1.49 | 0.70 | 1.12 | 0.96 | 0.68 | 1.02 | 1.85 | 0.95 | 1.14 | 1.12 | 1.73 |
| **D-Ser→D-Ala** | 1.05 | 44.10 | 31.74 | 26.58 | 14.34 | 26.20 | 63.92 | 11.48 | 15.89 | 28.87 | 36.21 | 30.21 | 27.63 | 26.07 | 18.59 | 23.83 | 58.29 |
| **D-Thr→D-Ala** | 0.94 | 30.60 | 31.66 | 18.60 | 8.67 | 24.91 | 61.35 | 9.40 | 12.44 | 19.28 | 23.20 | 24.91 | 13.01 | 15.84 | 14.51 | 62.31 | 45.11 |
| **D-Trp→D-Ala** | 1.00 | 118.57 | 102.50 | 76.08 | 33.43 | 69.70 | 194.45 | 37.71 | 42.60 | 70.75 | 65.72 | 98.43 | 63.09 | 49.49 | 74.70 | 24.69 | 179.81 |
| **D-Tyr→D-Ala** | 1.16 | 86.31 | 63.63 | 38.13 | 42.46 | 50.47 | 105.55 | nd | 37.09 | 46.94 | 45.37 | 91.34 | 36.63 | 56.35 | nd | 51.47 | 36.14 |
| **D-Val→D-Ala** | 0.94 | 39.88 | 35.01 | 27.65 | 16.23 | 39.71 | 77.92 | nd | 18.74 | 20.40 | 21.53 | 49.55 | 42.85 | 19.72 | 31.86 | 47.04 | 36.59 |

highlighted values: significantly decreased (in green) or increased (in red) ratio, nd: not determined

**Table S4:** Change of D-Glu ratio (compared to untreated control plants) after addition of D-AAs

|  | **Ler-0** | **Col-0** | **Bay-0** | **Cvi** | **Est-1** | **Kin-0** | **Nd-0** | **Van-0** | **Got-1** | **FR-2** | **Is-0** | **Nc-1** | **Nok-1** | **HR-5** | **C24** | **Shahdara** | **Ak-1** |
| --- | --- | --- | --- | --- | --- | --- | --- | --- | --- | --- | --- | --- | --- | --- | --- | --- | --- |
| **D-Ala→D-Glu** | 0.80 | nd | 37.93 | 20.26 | 5.59 | 11.07 | 33.56 | 10.92 | 13.37 | 80.58 | 11.88 | 9.03 | 19.84 | 8.61 | 14.34 | nd | nd |
| **D-Arg→D-Glu** | 0.87 | nd | 1.09 | 0.77 | 0.96 | 1.04 | 2.94 | 0.84 | 1.25 | 2.55 | 1.07 | 1.19 | 1.28 | 0.91 | 1.01 | nd | nd |
| **D-Asn→D-Glu** | 0.83 | nd | 11.14 | 0.91 | 1.03 | 2.78 | 10.14 | 1.87 | 2.38 | 24.65 | 2.53 | 1.58 | 1.65 | 1.56 | 2.91 | nd | nd |
| **D-Asp→D-Glu** | 0.95 | nd | 1.07 | 0.89 | 1.03 | 2.12 | 4.94 | 0.84 | 1.16 | 7.77 | 2.03 | 0.38 | 1.32 | 0.94 | 2.51 | nd | nd |
| **D-Gln→D-Glu** | 0.89 | nd | 23.20 | 24.05 | 4.85 | 10.90 | 30.29 | 7.72 | 8.01 | 30.64 | 7.20 | 3.40 | 7.34 | 5.09 | 9.22 | 35.55 | 18.57 |
| **D-Glu→D-Glu** | 185.12 | nd | 224.84 | 152.71 | 92.48 | 79.12 | 305.29 | 102.37 | 147.75 | 396.18 | 181.39 | 64.39 | 107.67 | 112.91 | 153.63 | 313.59 | 232.72 |
| **D-His→D-Glu** | 1.05 | nd | 13.90 | 10.37 | 0.94 | 5.56 | 17.90 | 4.67 | 3.78 | 22.50 | 4.84 | 4.76 | 3.77 | 2.80 | 3.44 | 10.81 | 6.54 |
| **D-Ile→D-Glu** | 1.02 | nd | 1.09 | 1.08 | 0.89 | 1.20 | 1.91 | 1.13 | 1.43 | 1.78 | 1.00 | 0.98 | 0.92 | 0.97 | 1.02 | 1.38 | 1.34 |
| **D-Leu→D-Glu** | 1.21 | nd | 6.57 | 6.86 | 1.08 | 3.96 | 15.09 | 3.83 | nd | 8.72 | 2.84 | 5.53 | 4.57 | 3.58 | 3.39 | 6.40 | 26.22 |
| **D-Lys→D-Glu** | 1.13 | nd | 1.54 | 0.92 | 1.00 | 0.94 | 3.84 | 0.82 | 1.27 | 1.80 | 1.00 | 1.71 | 0.00 | 0.91 | 0.97 | 1.82 | 5.91 |
| **D-Met→D-Glu** | 1.08 | nd | 23.20 | 14.17 | 5.20 | 7.07 | 36.12 | 11.24 | 9.10 | 58.44 | 9.46 | 9.76 | 8.75 | 8.45 | 11.35 | 18.13 | 33.05 |
| **D-Phe→D-Glu** | 1.20 | nd | 14.89 | 10.27 | 3.92 | 5.22 | 21.96 | 5.46 | 5.28 | 22.00 | 7.32 | 5.98 | 9.63 | 6.20 | 8.38 | 9.08 | 14.66 |
| **D-Pro→D-Glu** | 0.97 | nd | 1.10 | 0.80 | 1.05 | 0.91 | 1.51 | 0.70 | 1.12 | 0.96 | 1.00 | 0.73 | 1.40 | 0.94 | 1.14 | 2.78 | 1.73 |
| **D-Ser→D-Glu** | 1.05 | nd | 1.01 | 0.88 | 0.94 | 1.56 | 8.34 | 1.26 | 1.37 | 4.82 | 2.34 | 2.82 | 1.11 | 1.72 | 1.29 | 3.17 | 5.17 |
| **D-Thr→D-Glu** | 0.94 | nd | 1.17 | 0.88 | 0.98 | 1.54 | 3.83 | 0.83 | 1.19 | 2.61 | 1.14 | 2.13 | 0.68 | 1.00 | 0.88 | 7.50 | 4.48 |
| **D-Trp→D-Glu** | 1.00 | nd | 1.45 | 0.97 | 1.10 | 3.12 | 17.21 | 3.06 | 3.40 | 9.42 | 3.46 | 7.88 | 2.79 | 2.45 | 3.78 | 2.93 | 12.74 |
| **D-Tyr→D-Glu** | 1.16 | nd | 1.22 | 0.97 | 1.03 | 3.32 | 5.41 | nd | 2.22 | 5.61 | 1.33 | 5.21 | 1.11 | 3.46 | nd | 4.84 | 2.82 |
| **D-Val→D-Glu** | 0.94 | nd | 1.14 | 0.92 | 1.04 | 2.11 | 5.53 | nd | 1.18 | 2.26 | 1.27 | 3.55 | 1.20 | 1.24 | 2.16 | 4.75 | 3.02 |

highlighted values: significantly decreased (in green) or increased (in red) ratio, nd: not determined

**Figure S1**: Total ion chromatogram (TIC) showing up 34 analytes on the basis of 68 MRMs within 8 time segments (TS).


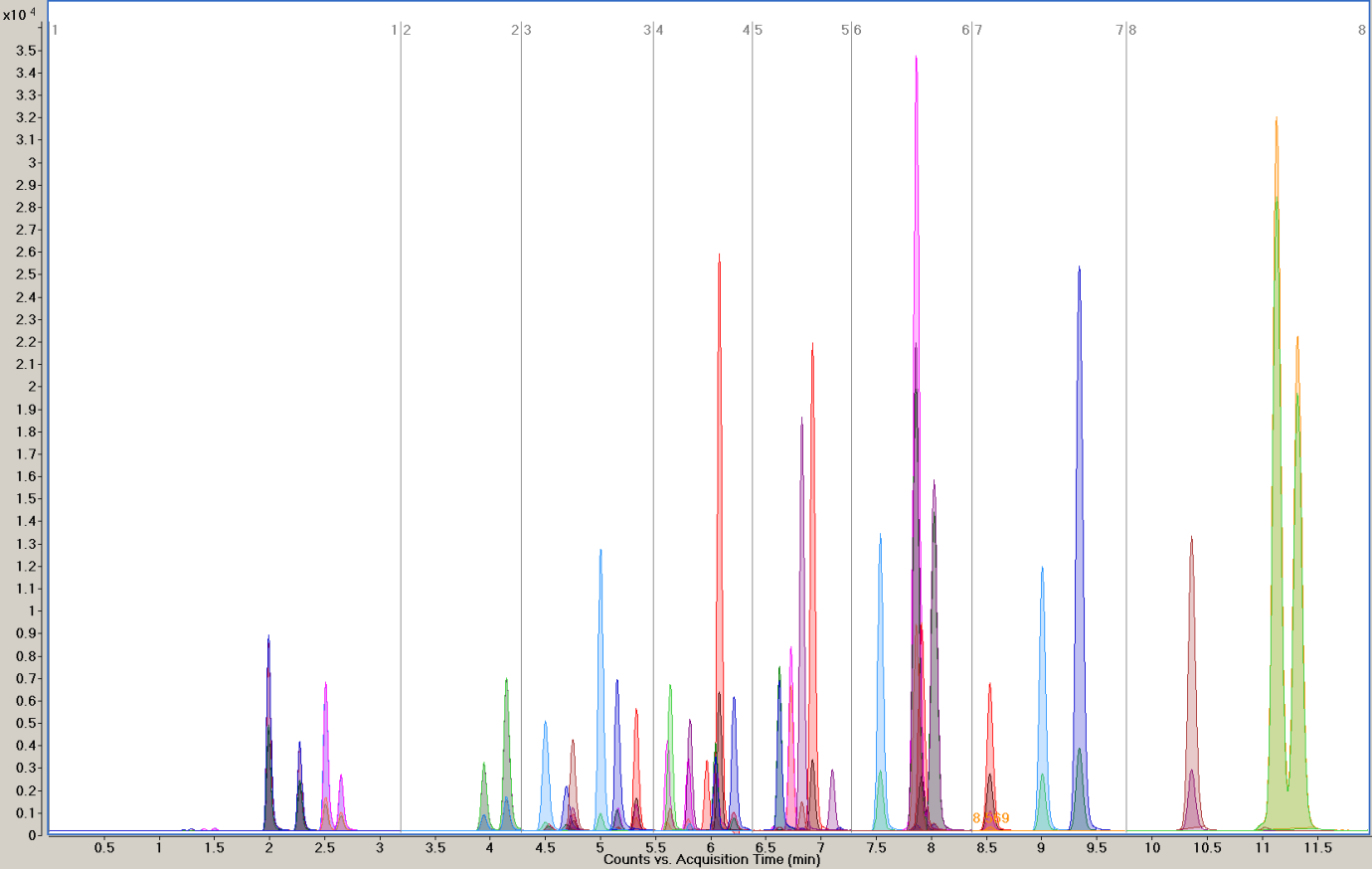


**Figure S2**: Representative calibration plot for D-Arg


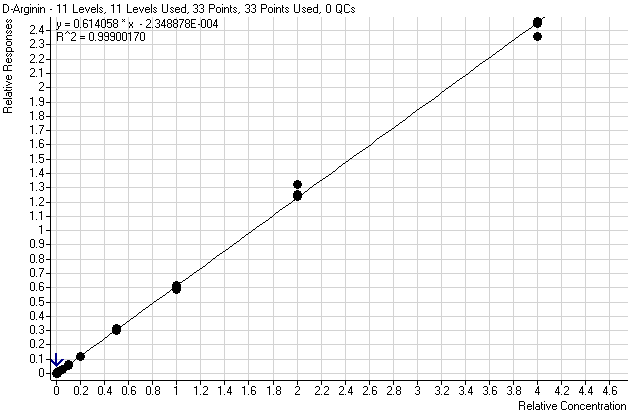


**Figure S3**: Transitions for AAs D-His, L-His, L-Asn, D-Asn, L-Ser, D-Ser, L-Ala, D-Ala, L-Met, D-Met, L-Ile, L-Leu, D-Ile, and D-Leu.


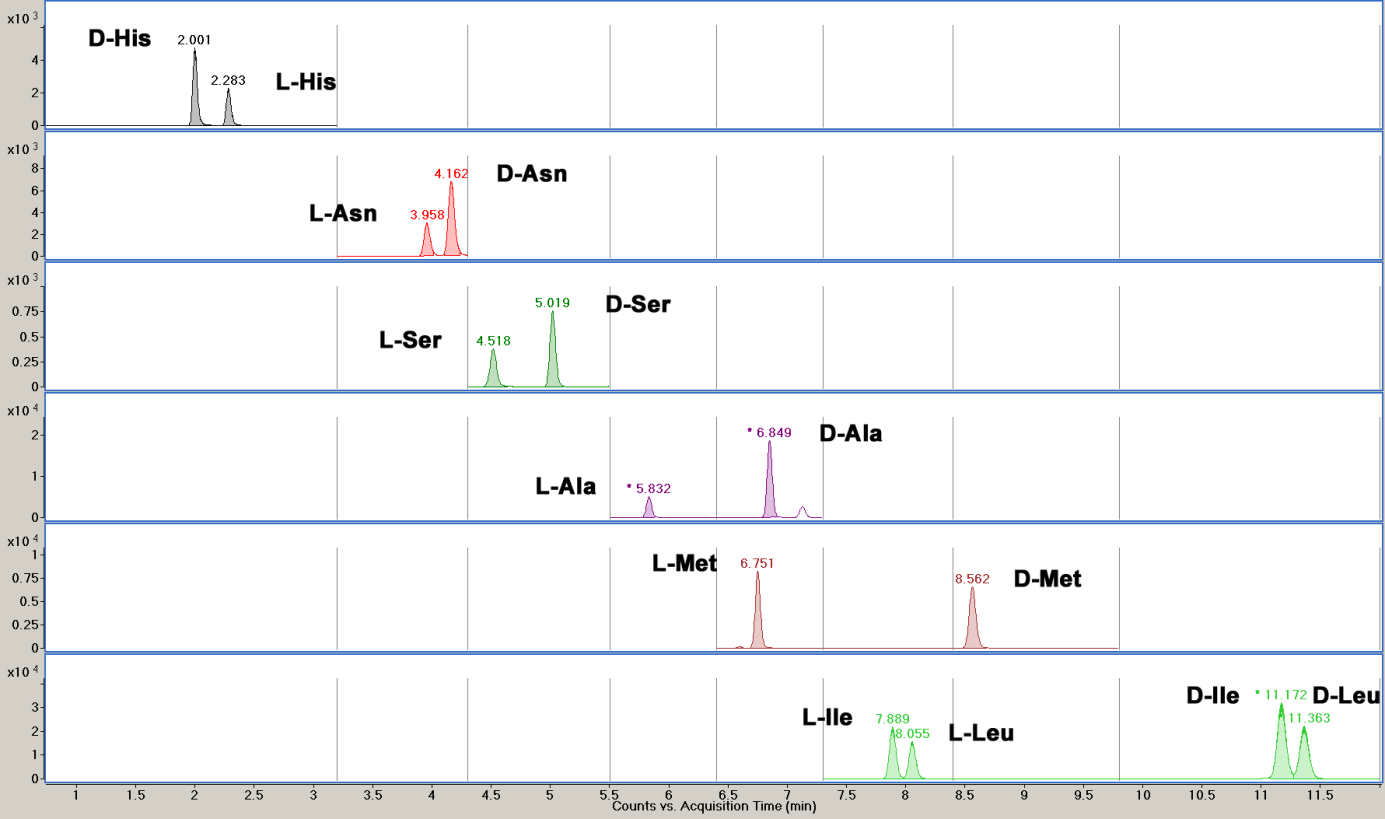


**Figure S4:** Transitions for AAs L-Asp, D-Asp, L-Thr, D-Thr, L-Glu, D-Glu, Gly, L-Val, D-Val, L-Trp, and D-Trp.


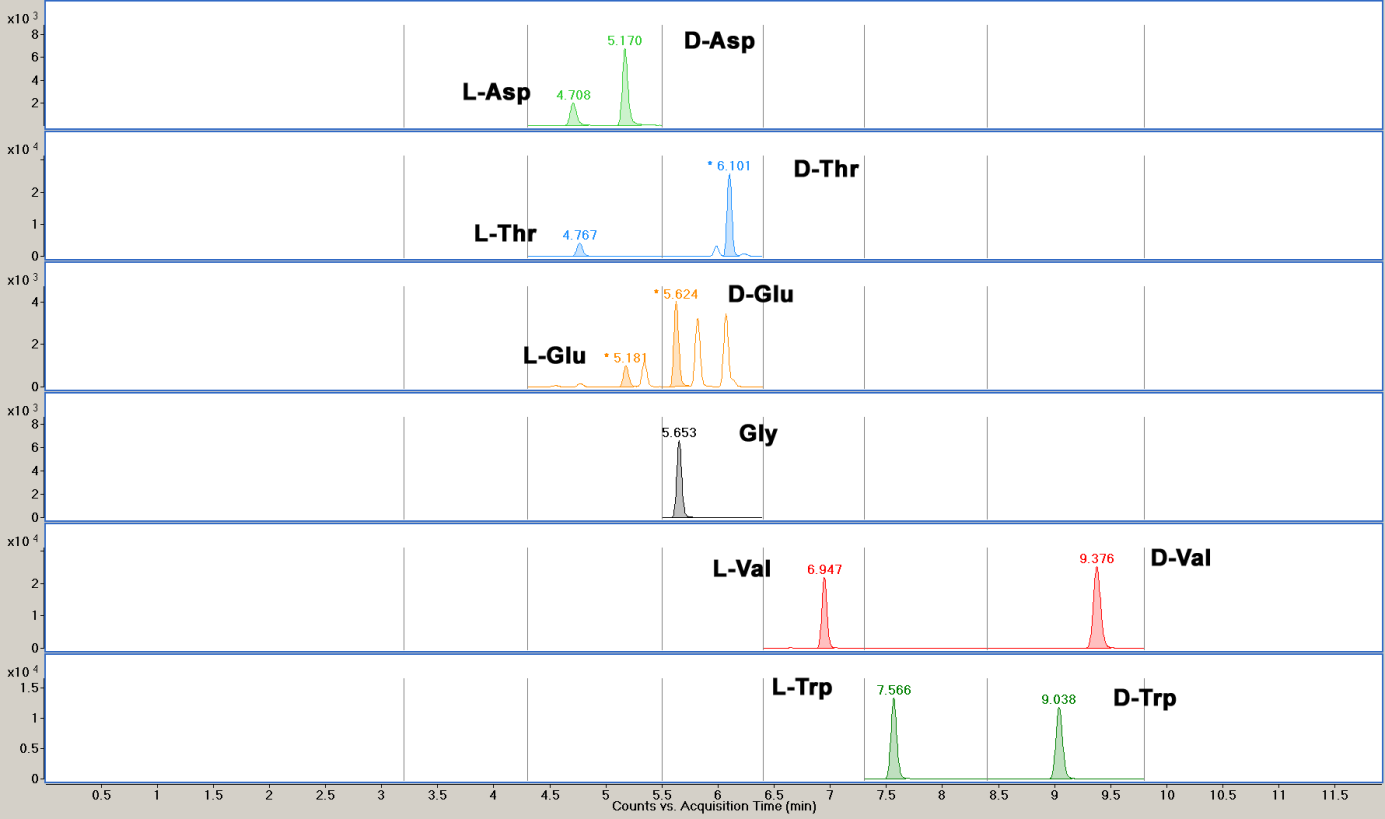


**Figure S5:** Transitions for AAs D-Arg, L-Arg, L-Gln, D-Gln, L-Pro, D-Pro, L-Phe d_8_ (internal standard), L-Phe, and D-Phe.


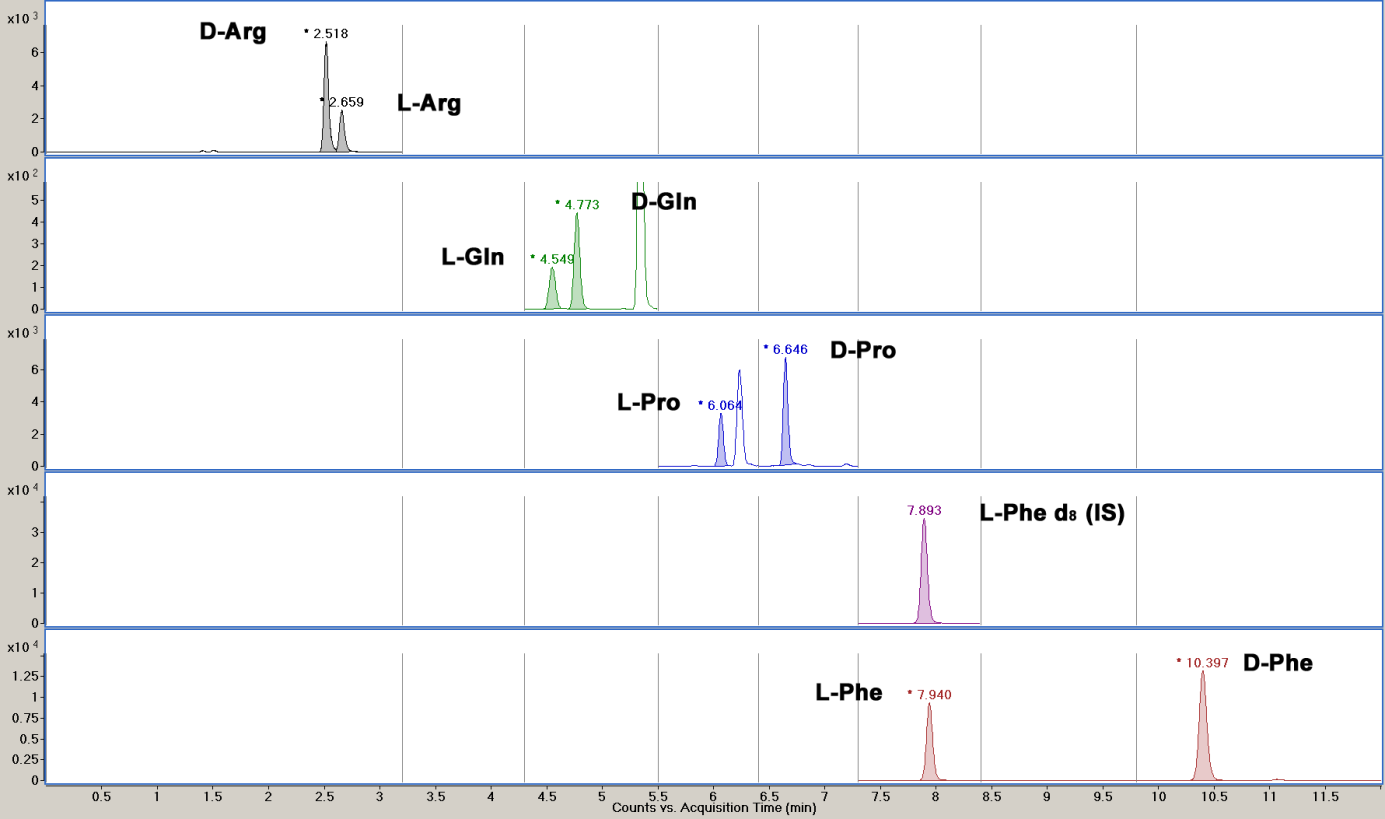

Supplement: Supplementary file 1 — Additional file 1: Table S1: Amino Acids Multiple Reaction Monitoring (MRM) Acquisition Parameters. Table S2: Slope, Correlation, and Limit of Quantitation for D- and L-AAs. Table S3: Change of D-Ala ratio (compared to untreated control plants) after addition of D-AAs. Table S4: Change of D-Glu ratio (compared to untreated control plants) after addition of D-AAs. Figure S1: Total ion chromatogram (TIC) showing up 34 analytes on the basis of 68 MRMs within 8 time segments (TS). Figure S2: Representative calibration plot for D-Arg. Figure S3: Transitions for AAs D-His, L-His, L-Asn, D-Asn, L-Ser, D-Ser, L-Ala, D-Ala, L-Met, D-Met, L-Ile, L-Leu, D-Ile, and D-Leu. Figure S4: Transitions for AAs L-Asp, D-Asp, L-Thr, D-Thr, L-Glu, D-Glu, Gly, L-Val, D-Val, L-Trp, and D-Trp. Figure S5: Transitions for AAs D-Arg, L-Arg, L-Gln, D-Gln, L-Pro, D-Pro, L-Phe d8 (internal standard), L-Phe, and D-Phe. (DOCX 323 KB) [file 40064_2013_614_MOESM1_ESM.docx]
